# An RSSI-Based Low-Power Vehicle-Approach Detection Technique to Alert a Pedestrian †

**DOI:** 10.3390/s20010118

**Published:** 2019-12-24

**Authors:** Yoshito Watanabe, Yozo Shoji

**Affiliations:** Social-ICT System Laboratory, National Institute of Information and Communications Technology, Koganei, Tokyo 184-8795, Japan; shoji@nict.go.jp

**Keywords:** edge computing, collision avoidance, traffic accident prevention, Student’s t-test, received signal strength indicator (RSSI)

## Abstract

Information about an approaching vehicle is helpful for pedestrians to avoid traffic accidents while most of the past studies related to collision avoidance systems have focused on alerting drivers and controlling vehicles. This paper proposes a technique to detect an approaching vehicle aiming at alerting a pedestrian by observing the variation of the received signal strength indicator (RSSI) of the repeatedly radiated beacons from a vehicle, called the alert beacons. A linear regression algorithm is first applied to RSSI samples. The decision about whether a vehicle is approaching or not is made by the Student’s *t*-test for the linear regression coefficient. A passive method, where the pedestrian’s device behaves only as a receiver, is first described. The neighbor-discovery-based (ND-based) method, in which the pedestrian’s device repeatedly broadcasts advertising beacons and the moving vehicle in the vicinity returns the alert beacon when it receives the advertising beacon, is then proposed to improve the detection performance as well as reduce the device’s energy consumption. The theoretical detection error rate under Rayleigh fading is derived. It is revealed that the proposed ND-based method achieves a lower detection error rate when compared with the passive method under the same delay.

## 1. Introduction

Japan is facing an aging society, and it is important to respect senior citizens and to promote the creation of a prosperous city for all people. Meanwhile, vehicles driven by elderly people are considered to be risk factors, and traffic accidents caused by them have become a serious social problem in Japan [1,2]. Although people over 70 years old should attach signs that indicate elderly drivers to their cars in Japan, the detection of these signs is not necessarily easy. Moreover, vehicles cannot be detected if they are in non-line-of-sight (NLoS) areas from pedestrians. Therefore, a system to alert pedestrians should be desirable for pedestrians, especially for children, who tend to engage in unpredictable behaviors, and their associated parents or teachers.

This paper proposes a technique to detect an approaching vehicle in order to alert a pedestrian, especially at a residential area where the visibility of roads is very limited. Assuming a pedestrian is carrying a beacon device, the detection technique is carried out by the received signal strength indicator (RSSI) of the repeatedly radiated beacons, called *alert beacons*, from the vehicle’s device under multipath environment. In the proposed system, the approach of the target is detected by the Student’s *t*-test for a linear regression coefficient, i.e., the slope of the successive RSSI samples. Since the above *passive method*, in which the pedestrian’s device behaves only as a receiver, consumes much battery energy by opening the receiver window all the time, we further propose the *neighbor-discovery-based (ND-based)* method. In the ND-based method, the beacon called an *advertising beacon*, is first transmitted from the pedestrian’s device. Then the vehicle’s device returns an alert beacon when it receives the advertising beacon. Since the proposed technique does not require additional functions such as cloud servers or infrastructural facilities, an alert system for pedestrians could be realized cost-effectively.

Also, the detection error rate of the proposed technique with given parameters is theoretically analyzed.

Please note that this work is a substantial extension of our initial work [3], in which the approach is detected only by the hard decision of the slope of RSSI samples with zero-thresholding in the framework of the passive method. Moreover, the detection was carried out at the vehicle side for a driver assistant system in the previous work. This paper employs a Student’s *t*-test to enhance the reliability of the system. Moreover, the ND-based method is proposed to improve the detection performance by doubling the number of RSSI samples and contributes to reducing the device’s energy consumption, assuming that an approaching vehicle is detected at the pedestrian side.

The rest of this paper is organized as follows. In Section 2, we review related work on collision avoidance systems and mobile speed estimation techniques. Section 3 gives an overview of the proposed system based on the passive method. Section 4 describes the proposed approach detection technique based on RSSI samples. Section 5 introduces the ND-based method to double the number of RSSI samples employed for the detection. Numerical evaluations of the detection performance are given in Section 6. Section 7 introduces our prototype devices, and Section 8 concludes this work.

## 2. Related Work

Vehicle-approach detection techniques based on radar scanner and visual imaging cameras are well-matured solutions for collision avoidance systems. There are a variety of proposals in the past literature, and they are well surveyed in [4,5,6]. Keller et al. [7] developed a vehicle control system that is integrated with pedestrian recognition by stereo vision trending to self-driving cars. Jeong et al. [8] proposed a method to detect a pedestrian during summer nights by a far-infrared (FIR) camera on a vehicle. Such techniques are useful for alerting an *emergency* hazard to the drivers. However, the application of the above systems is limited to situations where targets are directly visible from the vehicle. Moreover, there is a lack of a system that can alert a pedestrian side, not a vehicle side, in the literature.

TOYOTA has brought an intelligent transport system known as “ITS connect [9]” to avoid the traffic accidents with pedestrians in blind spots or at poor-visibility intersections. In such a system, pedestrians crossing roads are detected by specific devices deployed around the intersections, and that information is notified to drivers. However, it would be difficult to build these comparatively expensive infrastructural systems widely in residential streets. Considering that the situation where pedestrians such as children or elderly people are keeping some beaconing devices for safe and secure purposes becomes common, especially, in Japan, the proposed system is much more feasible in terms of cost-effectiveness.

There are proposals to estimate the mobile speed and the direction based on the Doppler frequency of received signals, e.g., in [10,11,12,13]. Still, they require the physical layer level implementations, and thus they are difficult to be implemented in practical applications. Since obtaining RSSI in the upper layer is relatively easy, the applications to localization systems have attracted much attention [14,15,16,17]. However, they seem to be still challenging because they need to deploy more than one access point to estimate the correct location of the targets as well as the exact channel propagation characteristics. Nevertheless, if the goal is limited to detect the target approach assuming the target is carrying any beaconing device, the system should be in more practical, and there is no need to estimate channel propagation characteristics in the proposed system.To the best of our knowledge, our work is the first trial to employ RSSI for the approach detection of vehicles and pedestrians.

Although the global positioning system (GPS) would be helpful to implement the functions mentioned above, the devices would be more costly, and the obtained precision would get low under building shadow. Moreover, the system employing a GPS tend to consume battery energy more quickly; the current consumption by a typical GPS module is about 14.4 mA, and thus the battery life of the device becomes only about 15 h if we employ a coin battery with the capacity of 220 mAh [18]. RSSI-based detection with the proposed ND-based method overcomes the issue of battery exhaustion, which will be described in Section 7.

## 3. System Model

### 3.1. Vehicle-Approach Detection Based on a Passive Method

**Figure 1:**
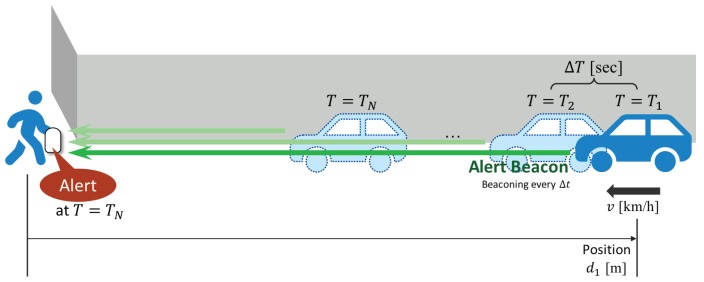
A system model of the proposed vehicle-approach detection technique.

Figure 1 illustrates the overall system model. A vehicle carries a device that radiates specific beacons, called *alert beacons*, repeatedly every ΔT seconds to notify its existence to the surroundings. A pedestrian carries a device that can receive the alert beacons. When the pedestrian receives an alert beacon, the detection of approaching begins.

**Figure 2:**
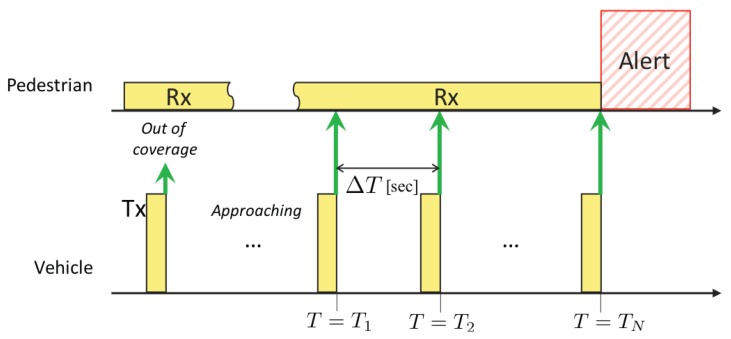
A sequence diagram of the approach detection based on the passive method.

The initial time, where the first beacon is received at the pedestrian, is denoted as $T=T_{1}$. It is assumed that the pedestrian keeps receiving the alert beacons in every ΔT seconds without packet loss, for simplicity. The timestamp of transmitting *n*-th advertising beacon is computed as

(1) $$T_{n}=T_{1}+\left(n-1\right)\Delta T$$

for n=1,2,⋯. After receiving *N* alert beacons in total, the pedestrian’s device estimates the approach of the vehicle at time $T=T_{N}$. If the system detects the approach, the system alerts the pedestrian by, e.g., voice navigation. In this approach, the pedestrian’s device behaves only as a receiver and keeps the receiver window open all the time, and thus we call it the *passive method*. A sequence diagram of the above process is shown in Figure 2.

### 3.2. Vehicle Motion Model

Let us denote the initial vehicle distance from the pedestrian at the time $T=T_{1}$ by $d=d_{1}$ m. For simplicity of analysis, it is assumed that the vehicle keeps moving with a *constant* velocity *v* km/h relatively to the pedestrian, where v<0 and v>0 represent receding and approaching, respectively. The receiving time of *n*-th beacon is denoted by $T_{n}$ for n=1,2,⋯ according to (1). Therefore, the *n*-th distance of the vehicle from the target is represented by

(2) $$d_{n}=d_{1}-v\left(T_{n}-T_{1}\right)=d_{1}-\left(n-1\right)v\Delta T.$$

### 3.3. RF Propagation Model

It should be a rare situation when the beaconing device is in Line-of-Sight (LoS) from the vehicle, and the propagation between them should be in NLoS in most cases. Then we need to consider multipath propagation.

This paper assumes that the propagation model is specified by the simplified path loss model with Rayleigh fading. With an attenuation factor κ and a path loss exponent λ, the average received power at the distance *d* is computed by

(3) $$\Omega \left(d\right)=\kappa d^{-\lambda },$$

where the reference distance is assumed to be 1 m. Then the probability density function (PDF) of the received *instantaneous* power $\dot{p}$ conditioned by the distance *d* under the Rayleigh fading conforms to the exponential distribution [19] in watts as

(4) $$f_{\dot{p}}\left(\dot{p}|d\right)=\frac{1}{\Omega (d)}\exp\left(-\frac{\dot{p}}{\Omega (d)}\right).$$

We further transform the fading model in decibels by employing a variable $p=10\log_{10}\left(\dot{p}\right)$ and obtain the following Gumbel-wise distribution:

(5) $$f_{p}\left(p|d\right)=\frac{10^{p/10}}{10\Omega \left(d\right)\log_{10}e}\exp\left(-\frac{10^{p/10}}{\Omega (d)}\right).$$

In this paper, we assume that any two channels that beacons pass through are uncorrelated.

### 3.4. Problem Formulation

Let us define the following two events:

(6) $$\begin{array}{l} H_{0}:v\leq 0\\ H_{1}:v>0, \end{array}$$

where in other words, in the case of $H_{1}$, the vehicle and the pedestrian are approaching each other.

We now denote the sequence of RSSI samples by $p=\{p_{1},\ldots ,p_{N}\}$, where *N* is the total number of RSSI of beacons. Our goal is to estimate the event from p. However, since $p_{n}$ for 1,…,N is by nature random variable conforming to the PDF (4), the detection of the event is not straightforward.

Let *x* and *y* denote the true event and the estimated event, respectively, where $x,y\in \{H_{0},H_{1}\}$. Considering the purpose of our application, the error probability $p(y=H_{0}|x=H_{1})$ should be as low as possible, accepting some degree of the error probability $p(y=H_{1}|x=H_{0})$, in which we call the former error a Type-I error and the latter a Type-II error. To this end, we employ a detection method based on a Student’s *t*-test, which is described in the following section.

The *detection delay* is another concern for the proposed system. Assuming that the computational time for detection is negligible, the detection delay can be computed as

(7) $$T_{\mathrm{delay}}=T_{N}-T_{1}=\left(N-1\right)\Delta T\;\left[s\right]$$

where in the case of the passive technique. From (7), we can expect that the larger ΔT and *N* tend to produce larger delay, which causes the severe approach of the vehicle and the pedestrian when $x=H_{1}$. We will analyze the trade-off between the detection performance and the detection delay, and derive appropriate setups for practical use.

## 4. A Vehicle-Approach Detection Technique Based on a Student’s *t*-Test

This section describes the proposed detection technique of the target approach based only on RSSI samples and their receiving times.

### 4.1. Linear Regression of RSSI Samples

We first approximate the RSSI sequence p as a linear function of time *t* with coefficient vector $\beta =\{\beta_{1},\beta_{2}\}$, which is represented by

(8) $$\phi \left(T\right)=\beta_{1}+\beta_{2}T.$$

To estimate coefficients β, we employ a least-squares method (LSM).

Let us define a population regression equation by

(9) $$p_{n}=\beta_{1}+\beta_{2}T_{n}+\varepsilon_{n},$$

where $\varepsilon_{n}$ is the error term at the time $T_{n}$ for n=1,…,N, or, Equation (9) can be written as

(10) $$\varepsilon_{n}=p_{n}-\left(\beta_{1}+\beta_{2}T_{n}\right),$$

for $\varepsilon_{n}$, the conditions that the mean $E\{\varepsilon_{n}\}=0$ and $E\{\varepsilon_{n}\varepsilon_{m}\}=0$ for n≠m are satisfied if $\varepsilon_{n}$ and $\varepsilon_{m}$ are uncorrelated with each other, respectively, and let us denote the variance of $\varepsilon_{n}$ by $V\left\{\varepsilon_{n}\right\}=\sigma_{n}^{2}$.

By employing an LSM for $\sum_{n=1}^{N}\varepsilon_{n}^{2}$, we can obtain the regression coefficients such that

(11) $${\hat{\beta }}_{2}=\frac{\sum_{n=1}^{N}\left(T_{n}-\bar{T}\right)\left(p_{n}-\bar{p}\right)}{\sum_{n=1}^{N}{\left(T_{n}-\bar{T}\right)}^{2}}$$

(12) $${\hat{\beta }}_{1}=\bar{p}-{\hat{\beta }}_{2}\bar{T},$$

where $\bar{T}$ and $\bar{p}$ are the sample means of $T_{n}$ and $p_{n}$ for n=1,…,N, respectively.

One can notice that the events $H_{0}$ and $H_{1}$ defined in (6) are equivalent to the conditions where $\beta_{2}\leq 0$ and $\beta_{2}>0$, respectively. Therefore, we analyze the statistical property of ${\hat{\beta }}_{2}$ in what follows.

### 4.2. Sample Distribution of ${\hat{\beta }}_{2}$

For given system parameters *N* and ΔT and any variables *v*, $d_{1}$, κ and λ, let us now assume that each error term $\varepsilon_{n}$ in Equation (9) for n=1,…,N is i.i.d. and conforms to the Gaussian distribution, i.e.,

(13) $$\varepsilon_{n}∼N\left(0,\sigma^{2}\right)\;\mathrm{for}\;n=1,\ldots ,N,$$

where the variance is assumed to be common as $\sigma^{2}=\sigma_{n}^{2}$ for all *n*.

Substituting Equation (9) into Equation (11), we can obtain that

(14) $${\hat{\beta }}_{2}=\beta_{2}+\frac{\sum_{n=1}^{N}\left(T_{n}-\bar{T}\right)\varepsilon_{n}}{\sum_{n=1}^{N}{\left(T_{n}-\bar{T}\right)}^{2}},$$

which shows that ${\hat{\beta }}_{2}$ can be represented as the linear function of Gaussian variables $\varepsilon_{n}$, and thus ${\hat{\beta }}_{2}$ also conforms to the Gaussian distribution. From $E\{\varepsilon_{n}\}=0$ and $E\{\varepsilon_{n}\varepsilon_{m}\}=0$ for n≠m, it follows that

(15) $$E\{{\hat{\beta }}_{2}\}=\beta_{2}$$

(16) $$V\{{\hat{\beta }}_{2}\}=E\left\{{\left({\hat{\beta }}_{2}-\beta_{2}\right)}^{2}\right\}=\frac{\sigma^{2}}{\sum_{n=1}^{N}{\left(T_{n}-\bar{T}\right)}^{2}}$$

and thereby

(17) $${\hat{\beta }}_{2}∼N\left(\beta_{2},\frac{\sigma^{2}}{\sum_{n=1}^{N}{\left(T_{n}-\bar{T}\right)}^{2}}\right).$$

Please note that for the observed RSSI $p_{n}$ and its regression value ${\hat{p}}_{n}=\hat{\phi }\left(T_{n}\right)={\hat{\beta }}_{1}+{\hat{\beta }}_{2}T_{n}$, the residual ${\hat{\varepsilon }}_{n}≜p_{n}-{\hat{p}}_{n}$ satisfies the following conditions:

(18) $$\sum_{n=1}^{N}{\hat{\varepsilon }}_{n}=0,\;\sum_{n=1}^{N}{\hat{\varepsilon }}_{n}T_{n}=0.$$

Since $\sigma^{2}$ in (17) is unknown in practice, we calculate the sample variance of $\varepsilon_{n}$ from the observed RSSI samples as follows:

(19) $$s^{2}=\frac{\sum_{n=1}^{N}{\hat{\varepsilon }}_{n}^{2}}{N-2},$$

where the denominator N−2 stems from the reduction of degrees of freedom due to the conditions in (18).

By replacing $\sigma^{2}$ in (17) with $s^{2}$, we can obtain the sample variance of ${\hat{\beta }}_{2}$, and thus the sample standard deviation can also be calculated as

(20) $$\xi \left({\hat{\beta }}_{2}\right)=\sqrt{\frac{s^{2}}{\sum_{n=1}^{N}{\left(T_{i}-\bar{T}\right)}^{2}}}.$$

We now introduce the following variable:

(21) $$\rho_{\beta_{2}}=\frac{{\hat{\beta }}_{2}-\beta_{2}}{\xi ({\hat{\beta }}_{2})}.$$

Because the standardized value of ${\hat{\beta }}_{2}$ can be represented by $\left({\hat{\beta }}_{2}-\beta_{2}\right)/\sqrt{\sigma^{2}/\sum_{n=1}^{N}{\left(T_{n}-\bar{T}\right)}^{2}}$ that conforms to the standard Gaussian distribution, the variable $\rho_{\beta_{2}}$ can be regarded as the value of ${\hat{\beta }}_{2}$ standardized by the sample standard deviation $\xi ({\hat{\beta }}_{2})$ and shall conform to the Student’s *t*-distribution with N−2 degrees of freedom.

### 4.3. Student’s *t*-Test

With the variable $\rho_{\beta_{2}}$, we employ a Student’s *t*-test to decide *y*. Let us denote the cumulative distribution function (CDF) of the Student’s *t*-distribution with ν degrees of freedom for ρ by T(ρ|ν)=q, and its inverse function can be expressed as

(22) $$\rho =T^{-1}\left(q|\nu \right)=\left\{\rho :\;T\left(\rho |\nu \right)=q\right\}$$

Since our interest is to detect whether v>0 or not, by substituting $\beta_{2}=0$ to (21), we can obtain

(23) $$\rho_{0}=\frac{{\hat{\beta }}_{2}}{\xi ({\hat{\beta }}_{2})}.$$

With a significance level $\alpha_{0}$ for v=0, if the condition $\rho_{0}\geq T^{-1}\left(\alpha_{0}|N-2\right)$ is satisfied, we decide as $y=H_{1}$, otherwise $y=H_{0}$.

Please note that in practice, $T^{-1}\left(\alpha_{0}|N-2\right)$ can be computed by referring to the predefined Student’s *t*-distribution table, and thus, its computational complexity is negligible. We also note that the value of the significance level $\alpha_{0}$ directly affects the detection performance, and in the case where $\alpha_{0}=0.5$, the above Student’s *t*-test is equivalent to the simple decision making that has been presented in our previous work [3], where $y=H_{1}$ if ${\hat{\beta }}_{2}>0$ and $y=H_{0}$ if ${\hat{\beta }}_{2}\leq 0$ since $T^{-1}\left(0.5|\nu \right)=0$ for any ν.

### 4.4. Summary of the Detection Flow

The detection of the approaching from the observed RSSI samples p is summarized as follows:

1. compute $T^{-1}\left(\alpha_{0}|N-2\right)$ beforehand by the Student’s *t*-distribution table,
2. estimate ${\hat{\beta }}_{2}$ by (11),
3. compute $s^{2}$ and $\xi ({\hat{\beta }}_{2})$ by (19) and (20),
4. compute $\rho_{0}$ by (23), and
5. if $\rho_{0}\geq T^{-1}\left(\alpha_{0}|N-2\right)$, $y=H_{1}$, otherwise $y=H_{0}$.

## 5. ND-Based Vehicle-Approach Detection

We have mentioned that there is a trade-off between the detection performance and the detection delay. The latter directly stems from the number of RSSI samples employed in the approach detection for a specific ΔT. This section describes a new scheme that can double the number of RSSI samples by applying the *ND-based method* to the approach detection scenario.

**Figure 3:**
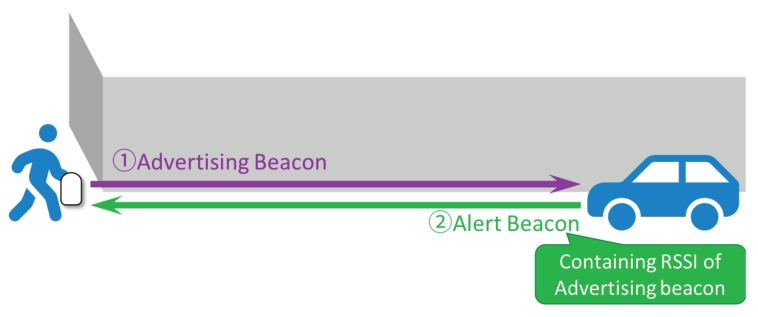
The proposed ND-based method.

Figure 3 illustrates the proposed ND-based method. Unlike the passive method, the pedestrian’s device first transmits specific beacons, called *advertising beacons*, repeatedly in every ΔT seconds.

Once a vehicle receives an advertising beacon, it returns an alert beacon containing the RSSI value of the advertising beacon inside the payload of the packet. The vehicle returns alert beacons to the pedestrian side every time it receives advertising beacons.

We now denote that the total number of advertising beacons by *M*, which is equivalent to that of alert beacons. Since we can employ the RSSI values of both the advertising beacons and the alert beacons at the pedestrian side, the total number of RSSI samples can be doubled such that N=2M.

**Figure 4:**
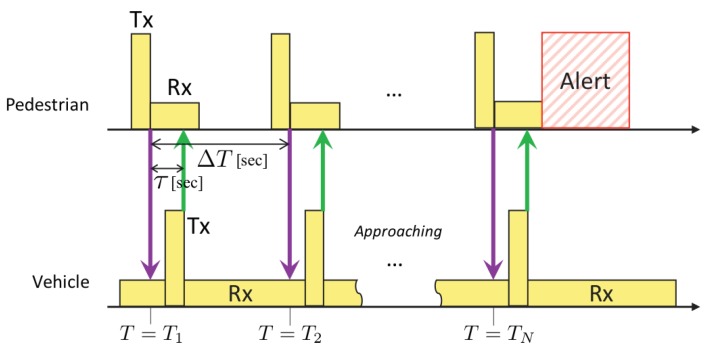
A sequence diagram of the ND-based system.

The initial time $T_{1}$, in this case, is defined by the time when a vehicle receives an advertising beacon for the first time. The response lag from the reception of an advertising beacon to the transmission of an alert beacon is denoted by τ in seconds. The timestamp of the RSSI sample $p_{n}$ can be represented by

(24) $$T_{n}=\left\{\begin{array}{ll} T_{1}+\left(m-1\right)\Delta T & \mathrm{for}\;n=2m-1\\ T_{1}+\left(m-1\right)\Delta T+\tau & \mathrm{for}\;n=2m \end{array}\right.$$

for m=1,2,⋯,M. After receiving *M* alert beacons responding to *M* advertising beacons, the detection finishes at the time $T_{N}=T_{2M}$. If the system detects an approaching vehicle, the system alerts the pedestrian. A sequence diagram of the above process is shown in Figure 4.

The ND-based method also has the advantage of prolonging the battery life of the pedestrian’s device compared to the passive method. According to [18], *instantaneous* current consumption per beacon transmission is about 94.4$\times 10^{-3}$ mAsec while the reception process *continuously* consumes 5.4 mA, which is more dominant than that of the transmission process if the reception process lasts long. In the ND-based method, the device can open the receiver window only while it waits for an alert beacon such that we can limit the duration time of the reception process. As a consequence, the battery life of the ND-based method is seven times longer than that of the passive method. A more detailed analysis of the power consumption can be seen in [18]. Although the ND-based method has a secondary advantage that we can also alert the driver side about the approaching pedestrian, this topic is beyond the scope of this paper.

## 6. Results

In practice, $d_{1}$ and *v* are random variables. The final goal of this study is to suitably design system parameters ΔT, τ, *N* and $\alpha_{0}$ to accommodate typical values of $d_{1}$ and *v*. This section shows numerical results on several scenarios of $d_{1}$ and *v*.

**Figure 5:**
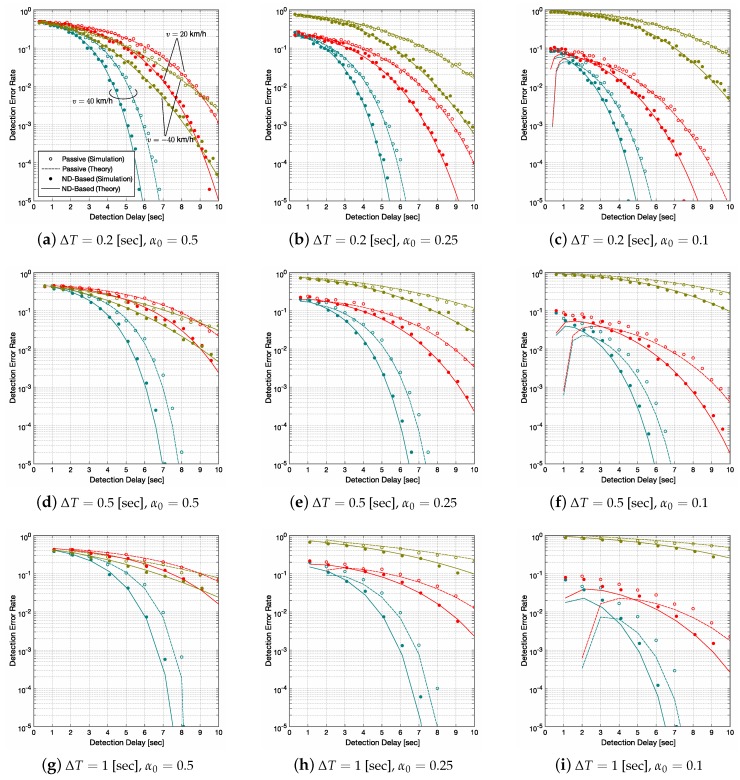
Detection delay vs. detection error rate. The detection error rates for v=40 and 20 km/h show the probability of Type-I errors and those for v=−40 km/h show the probability of Type-II errors.

Figure 5 shows the relationship between the detection error rate and the detection delay $T_{\mathrm{delay}}$ for both passive and ND-based methods with any combinations of system parameters ΔT=0.2, 0.5 and 1 s and $\alpha_{0}=0.5$, 0.25 and 0.1. Each figure includes the results for the vehicle velocities v= 40, 20 and −40 km/h with $d_{1}=100$ m. These parameters stem from the legal speeds for vehicles in residential areas and the communication range of beacons [18]. The parameters related to the propagation model are set as $\kappa =1\times 10^{-3}$ and λ=2.35, and the response lag τ in the ND-based method is set as τ=0.1 s. The simulation values are obtained by a Monte Carlo method, in which RSSI samples are generated randomly according to the PDF (5). We also derived the theoretical error rate, which is described in the Appendix A. The difference between the simulation values and the theoretical curves stems from the assumption in (13), i.e., the mismatch between the true distribution of $\varepsilon_{n}$ and the Gaussian distribution. Please note that the results for v=40 km/h and 20 km/h show the probability of Type-I errors and those for v=−40 km/h show the probability of Type-II errors.

It can be first shown in Figure 5 that the detection error rate decreases as $T_{\mathrm{delay}}$ increases in all cases; the performance is improved as the value of *N* increases for any combination of ΔT and $\alpha_{0}$. However, we should remember that the more $T_{\mathrm{delay}}$ increases, the closer the vehicle approaches to the pedestrian when $x=H_{1}$. It is also shown that the ND-based method is effective for reducing the detection error rate, and the detection delay can be shortened by almost one second to achieve the error rate of $10^{-3}$ in all cases.

When we compare the different values of $\alpha_{0}$ for a fixed ΔT, e.g., Figure 5a–c, a trade-off can be seen between the probabilities of Type-I errors and Type-II errors; Type-I errors reduce as $\alpha_{0}$ decreases while Type-II errors increase. For variable ΔT with a fixed $\alpha_{0}$, e.g., Figure 5a,d,g, as ΔT increases, the detection performance is degraded for all *v*.

From the results in Figure 5, one can see that the ND-based method with ΔT=0.2 s is the best option among the others, but the pedestrian’s device highly consumes battery energy in such a system. On the other hand, the system with ΔT=1 s increases the detection delay when compared to the same detection error rate level. For example, in the case that v=40 km/h, the ND-based method with ΔT=0.2 and $\alpha_{0}=0.5$ achieves the detection error rate of $10^{-2}$ at $T_{\mathrm{delay}}≃4.5$ s, in which the distance between the vehicle and the pedestrian is $d_{N}≃50$ m. To achieve the equivalent level of the above detection error rate, $T_{\mathrm{delay}}$ of 5.6 and 6.1 s are required for ΔT=0.5 s and 1 s.

**Figure 6:**
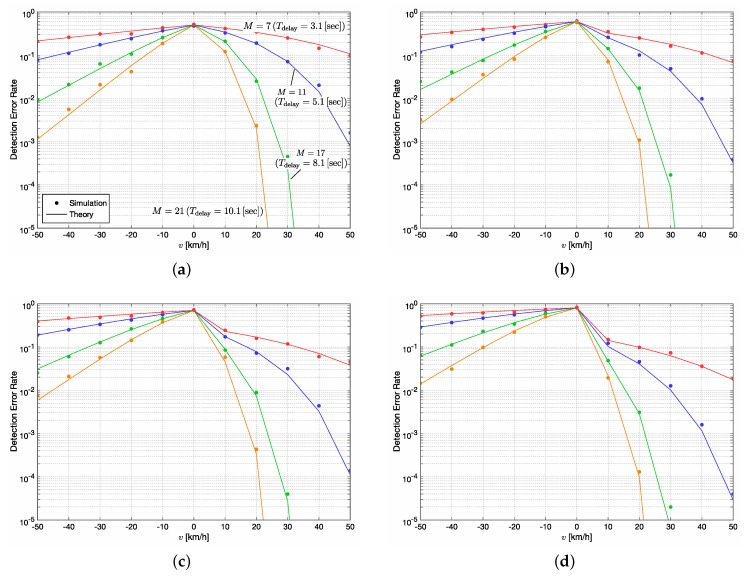
Detection error rate vs. vehicle velocity *v* for the ND-based method with ΔT=0.5 s and various *M*. (**a**) $\alpha_{0}=0.5$; (**b**) $\alpha_{0}=0.4$; (**c**) $\alpha_{0}=0.3$ and (**d**) $\alpha_{0}=0.2$. The detection error rates for v>0 and v≤0 respectively correspond to the probabilities of Type-I errors and Type-II errors.

Based on the above observations, we analyze the ND-based method with ΔT=0.5 s more in detail in what follows. Figure 6 illustrates the relationship between the detection error rate and the vehicle velocity *v* for the ND-based method with ΔT=0.5 s and various *M*, i.e., M=7, 11, 17 and 21, which correspond to $T_{\mathrm{delay}}=3.1$, 5.1, 8.1 and 10.1 s, respectively. The significance level $\alpha_{0}$ is set as 0.5, 0.4, 0.3 and 0.2 for Figure 6a–d respectively. We again note that the results for v>0 and v≤0 respectively correspond to the probabilities of Type-I errors and Type-II errors. As designed, we can see the detection error rate, or the probability of Type-II errors, for v=0 km/h is equal to $1-\alpha_{0}$.

The asymmetry of curves around the boundary of v=0 km/h is observed in all figures. It is because the mean of RSSI varies in $\log_{10}$ order with respect to the distance *d* as shown in (A1) while *d* changes linearly. Comparing Figure 6b to Figure 6a, i.e., $\alpha_{0}=0.4$ to 0.5, the detection performance for $x=H_{1}$ is improved. The effectiveness is much obvious around v=10 km/h, while some degradation of the performance for $x=H_{0}$, i.e., the increase of the probability of Type-II errors, can be seen. This degradation is more significant as $\alpha_{0}$ decreases as shown in Figure 6c,d. For example, when v=−40 km/h, the detection error rate for M=17 is less than $10^{-1}$ in Figure 6a–c, but it is almost equal to $10^{-1}$ in Figure 6d.

We now assume $10^{-2}$ is the allowable detection error rate for v=40 km/h. If we consider M=11, the techniques with $\alpha_{0}=0.4$, 0.3 and 0.2 can achieve the detection error rate of $10^{-2}$. However, as we decrease $\alpha_{0}$, the probability of Type-II errors increases, as mentioned above. In this case, the ND-based method with $\alpha_{0}=0.4$ becomes an appropriate design.

It turns out that it is challenging to determine *M* to a unique value since the performance fluctuates depending on the random variable *v* and $d_{1}$. Nevertheless, the design of a stepwise detection and alert system could be still possible with several predefined values of *M*, which would be the most feasible and reliable application for our scenario.

## 7. Prototype Devices

**Figure 7:**
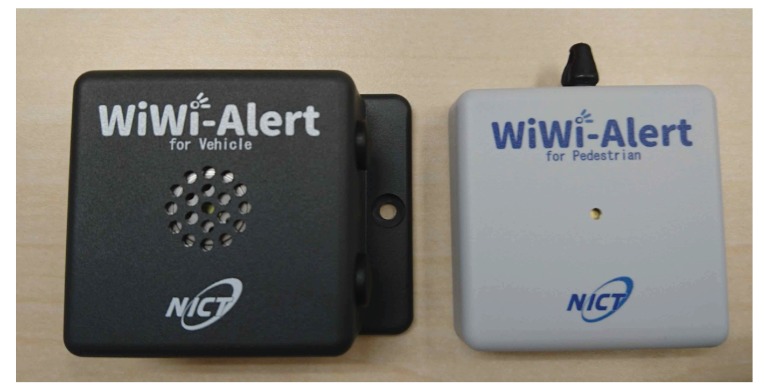
The prototype devices for a vehicle (**left**) and a pedestrian (**right**). The size of both devices is 5 cm × 5 cm

Figure 7 shows the prototype devices that we have developed for the proposed system. The wireless standard of IEEE 802.15.4g [20] is employed for the beacons. Both devices have a micro-processing unit (MPU) such that we can implement and conduct the proposed technique inside these devices. The vehicle’s device has a GPS, and thus we can add a function to reduce the transmission of unnecessary beacons when the velocity is below a predefined threshold. Likewise, the pedestrian’s device has an accelerometer such that the timing of beacon transmission can be controlled by recognizing the pedestrian’s action.

Our preliminary experiments [18] revealed that over 100 m of the beacon transmission range could be promising, and thus the proposed detection technique is feasible by the prototype devices. Each device has a unique medium access control (MAC) address. Therefore, the proposed system can distinguish the beacons of an identical device from those of other devices even in the case that there are several devices in the same area.

Rechargeable batteries with a capacity of 400 mAh are installed on both devices. If we assume the battery capacity is 220 mAh and the device is active for two hours per 24 h, about 280 days of battery life is attained by the ND-based method [18]. Therefore, it turns out that over 500 days of battery life is achievable by these prototype devices without recharging.

This paper focuses on the analysis of the proposed technique from the theoretical aspect to validate its feasibility. Further analysis based on real-field experiments with the prototype devices is beyond the scope of this paper.

## 8. Conclusions

This paper proposed an RSSI-based low-power vehicle-approach detection technique that alerts neighboring pedestrians. There are two approaches. A passive method applies a simple linear regression to estimate the slope of the RSSI samples and conducts a Student’s *t*-test from a limited number of received packets.

This paper also proposed the ND-based method that can significantly improve the detection performance by increasing the number of RSSI samples employed for the detection and reduce the power consumption of the pedestrian’s device.

The main findings of this paper are: (1) The ND-based method can shorten the detection delay by one second compared to the passive method to achieve the error rate of $10^{-3}$ in all cases. (2) There is a trade-off between the Type-I error rate and the Type-II error rate depending on the significance level in the Student’s *t*-test. (3) The ND-based method with the significance level $\alpha_{0}=0.4$ and the beacon interval ΔT=0.5 s seems to be an appropriate design in the simulations, and the stepwise detection could be the most reasonable implementation for our scenario.

The proposed technique does not require any prior knowledge, such as the speed of vehicles, the distance between the pedestrian and the vehicle, and the channel information for beacons. Therefore, the proposed technique can be adapted to situations where these parameters randomly vary. The analysis for more realistic conditions and the evaluation of actual detection performance in real-field experiments using the prototype devices are left as our future work.

## Appendix A. Derivation of Theoretical Error Rate

This appendix derives the theoretical detection error rate of the approach detection technique based on the Student’s *t*-test.

The mean of the PDF (5) at the distance *d* is expressed by

(A1) $$E\{p|d\}=10(\log_{10}\Omega \left(d\right)-\gamma \log_{10}e),$$

where γ is the Euler’s constant. The variance can be represented by

(A2) $$V\{p|d\}=\frac{50}{3}{\left(\pi \log_{10}e\right)}^{2},$$

which is constant regardless of the distance *d*.

We now conveniently denote the variance in (16) by $\varsigma^{2}≜V\left\{{\hat{\beta }}_{2}\right\}$. When $x=H_{1}$, the Type-I detection error rate can be represented by the probability where the system erroneously makes a decision as $y=H_{0}$, i.e., $\rho_{0}<T^{-1}\left(\alpha_{0}|N-2\right)$. Considering that $\rho_{0}$ is the estimated slope value scaled by the sample standard deviation $\xi ({\hat{\beta }}_{2})$, its statistical distribution conforms to

(A3) $$\rho_{0}∼N\left(\frac{\beta_{2}}{\sqrt{\varsigma^{2}}},1\right).$$

Consequently, the theoretical probability of Type-I errors can be computed by

(A4) $$\begin{array}{c} \mathrm{Pr}\left\{y=H_{0}|x=H_{1}\right\}=\mathrm{Pr}\left\{\rho_{0}<T^{-1}\left(\alpha_{0}|N-2\right)|\beta_{2}>0\right\}\\ \;\;\;\;=\frac{1}{2}\mathrm{erfc}\left(\frac{\beta_{2}-\sqrt{\varsigma^{2}}T^{-1}\left(\alpha_{0}|N-2\right)}{\sqrt{2\varsigma^{2}}}\right), \end{array}$$

where erfc(·) is the complementary error function. Likewise, the theoretical probability of Type-II errors can also be derived as

(A5) $$\begin{array}{c} \mathrm{Pr}\left\{y=H_{1}|x=H_{0}\right\}=\mathrm{Pr}\left\{\rho_{0}\geq T^{-1}\left(\alpha_{0}|N-2\right)|\beta_{2}\leq 0\right\}\\ \;\;=1-\frac{1}{2}\mathrm{erfc}\left(\frac{\beta_{2}-\sqrt{\varsigma^{2}}T^{-1}\left(\alpha_{0}|N-2\right)}{\sqrt{2\varsigma^{2}}}\right). \end{array}$$

With given system parameters *N* and ΔT, channel parameters κ and λ, and the vehicle-related variables *v* and $d_{1}$, we derive $\beta_{2}$ and $\varsigma^{2}$ in what follows.

The true slope $\beta_{2}$ can be estimated by calculating the regression coefficients of RSSI samples without any multipath effect. Since Equation (A1) represents the average power under the Rayleigh fading, the true slope $\beta_{2}$ can be calculated by

(A6) $$\beta_{2}=\frac{\sum_{n=1}^{N}\left(T_{n}-\bar{T}\right)\left(E\left\{p_{n}|d_{n}\right\}-\bar{E\{p_{n}|d_{n}\}}\right)}{\sum_{n=1}^{N}{\left(T_{n}-\bar{T}\right)}^{2}},$$

where $\bar{E\{p_{n}|d_{n}\}}$ is the sample mean of $E\{p_{n}|d_{n}\}$ for n=1,…,N.

We assume that the variance of the error term $\sigma^{2}$ is approximated to the variance (A2), i.e., $\sigma^{2}=V\left\{p|d\right\}$. Thereby, based on (16), we obtain

(A7) $$\varsigma^{2}=\frac{V\{p|d\}}{\sum_{n=1}^{N}{\left(T_{n}-\bar{T}\right)}^{2}}.$$
